# Fournier gangrene: rare complication of rectal cancer

**DOI:** 10.11604/pamj.2015.20.288.5506

**Published:** 2015-03-24

**Authors:** Pierlesky Elion Ossibi, Tarik Souiki, Karim Ibn Majdoub, Imane Toughrai, Said Ait Laalim, Khalid Mazaz, Somuah Tenkorang, My Hassan Farih

**Affiliations:** 1 Visceral Surgery Department, Hassan II University Hospital, Fez, Morocco; 2 Urology Department, Hassan II University Hospital, Fez, Morocco

**Keywords:** Fournier's Gangrene, rectal cancer, complication

## Abstract

Fournier's Gangrene is a rare complication of rectal cancer. Its discovery is often delayed. It's incidence is about 0.3/100 000 populations in Western countries. We report a patient with peritoneal perforation of rectal cancer revealed by scrotal and perineal necrotizing fasciitis.

## Introduction

Extra peritoneal perforation of rectal cancer is rare. It is often discovered late by severe perineal infection, requiring rapid diagnosis and urgent medical and surgical treatment. We report a case of a patient with extra peritoneal perforation of rectal cancer revealed by scrotal and perineal necrotizing fasciitis.

## Patient and observation

**Figure 1:**
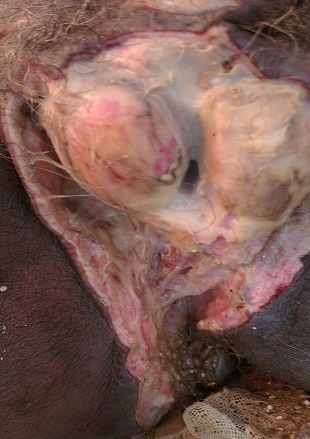
Fournier's gangrene

**Figure 2:**
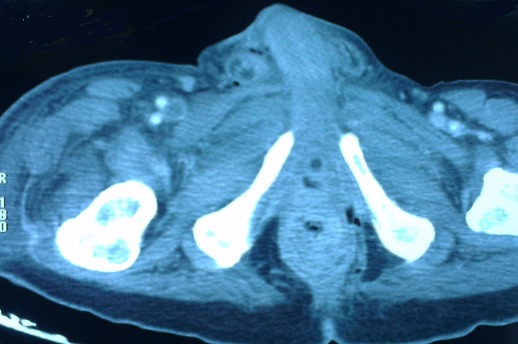
CT scan of the pelvis showing the tumor

**Figure 3:**
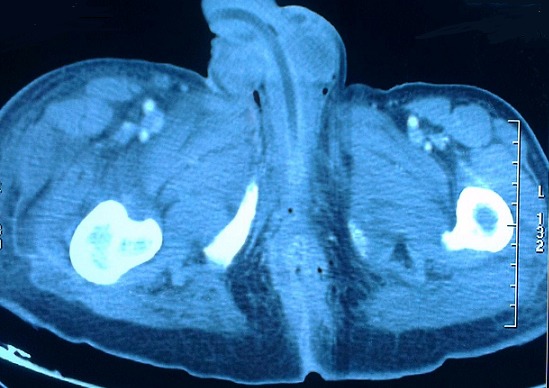
CT scan of the pelvis showing fasciitis with air bubbles

A 60 year old patient, who had been recently diagnosed of a non-metastatic adenocarcinoma of the rectum, presents with a painful swelling of the perineum and scrotum that had began 15 days prior to his admission, associated with fever and altered general condition. Physical examination found an altered, feverish patient, up to 39°C with a reddish, hot and painful swelling with areas of necrosis extending from the perineum to the root of the penis involving the scrotum (Figure 1). Digital rectal examination revealed a circumferential bulging tumor located 3 cm from the anal margin. Laboratory tests showed leukocytosis (18,900/mm^3^) and elevated C-reactive protein levels (697 mg/l). Abdominal and pelvic CT scan objectified an extra peritoneal perforated and necrotic huge rectal tumor (Figure 2) with a massive infiltration of the perineum containing air bubbles (Figure 3). However, there was no intra-peritoneal effusion found. The patient was put on broad spectrum antibiotics. Excision of necrotic tissues with an elective sigmoid colectomy was performed. Treatment of the rectal cancer will only be considered after complete control of the infection.

## Discussion

Fournier gangrene is a form of necrotizing fasciitis that affects the genitals, perineal and perianal region resulting from a polymicrobial infection whose source can be genitourinary, colorectal, skin or idiopathic, which could be potentially lethal [1]. The incidence of gangrene Fournier is about 0.3 / 100 000 in Western countries. In most reported cases, patient ages ranges between 30 and 60 years. A literature review in 1996 found 56 pediatric cases, of which 66% aged less than three months. [2] Men are ten times more affected than women [1–4]. This difference can be explained by better drainage of the perineal region in women through vaginal secretions. Although many cases described are idiopathic [5], its etiology is identified in 75-100% of patients. Its origin is colorectal in 13-50% of cases, urogenital in 17-87% of patients [3, 4]. Other causes include skin infections and local trauma. Colorectal sources include perirectal and perianal abscess, rectal instrumentation [6, 7], large bowel perforation due to colon cancer [8], diverticulitis [4], hemorrhoids [6] and anal intercourse among homosexuals.

Early diagnosis mainly depends on the vigilance of the clinician to suggestive symptoms and signs. However, the median time for diagnosis is elongated, six days on the average. Clinical diagnosis becomes evident when there is edema, crepitus, areas of dark red color moving rapidly towards extensive gangrene especially if they involve signs of severe sepsis Imaging can help diagnose the disease, its etiology and guide its treatment, but should not delay treatment: plain radiography and scrotal ultrasound may show air in the subcutaneous tissue before the onset of crepitus on clinical examination [9, 10]. The presence of air on x-ray, scanner or clinical examination is an absolute indication for urgent surgery. The scanner [11] and magnetic resonance imaging allow you to specify the limits of the infection and therefore determine the extent of debridement and eliminate the presence of deep skin abscesses.

This condition is an extreme emergency. It requires resuscitation and antibiotics and especially surgery. It is important to hospitalize these patients in an intensive care environment and to immediately initiate a probabilistic antibiotic active against anaerobes and Gram negative bacteria because of the potential severity of this infection and its progression to septic shock. Surgical treatment remains the most effective, irreplaceable, therapeutic measure [12] that determines the prognosis of this infection. Surgical treatment must be the fast associated with a possible hyperbaric oxygen therapy. Multidisciplinary management should be initiated without delay [13]. Surgical treatment involves debridement followed by regular wound dressings and skin reconstruction later. Bowel bypass is imperative in many cases. It must be performed early and during the initial phase [14]. In other cases, it is done when the peri-rectal area or the anal margin is threatened or feces threaten to contaminate infected lesions or incisions or drainage in fragile patients. Lack or late of realization of colostomy is identified in these cases, as a factor of poor prognosis or delay healing. The overall mortality varies greatly depending on centers from 5% to 45% in the current series. It is 16% in a series of 1726 cases published in 2000 and 7.5% in a series of 1641 men published in 2009 [13].

## Conclusion

Fournier gangrene is considered a major surgical emergency because of the importance of fasciocutaneous necrosis and its high mortality rate. This is a condition that has many causes including the extraperitoneal perforation of rectal cancer. Errors in diagnosis and inadequate treatment are caused as this disease remains not well known.
